# Supplementary material for: The causal relationship between the human gut microbiota and pyogenic arthritis: a Mendelian randomization study
Source: Front Cell Infect Microbiol. 2024 Nov 26;14:1452480. doi: 10.3389/fcimb.2024.1452480 (PMC11629706; doi:10.3389/fcimb.2024.1452480)
Supplement: Supplementary file 1 [file DataSheet1.zip › mendelian test/ebi-a-GCST90027619.csv_leave_one_out.pdf]

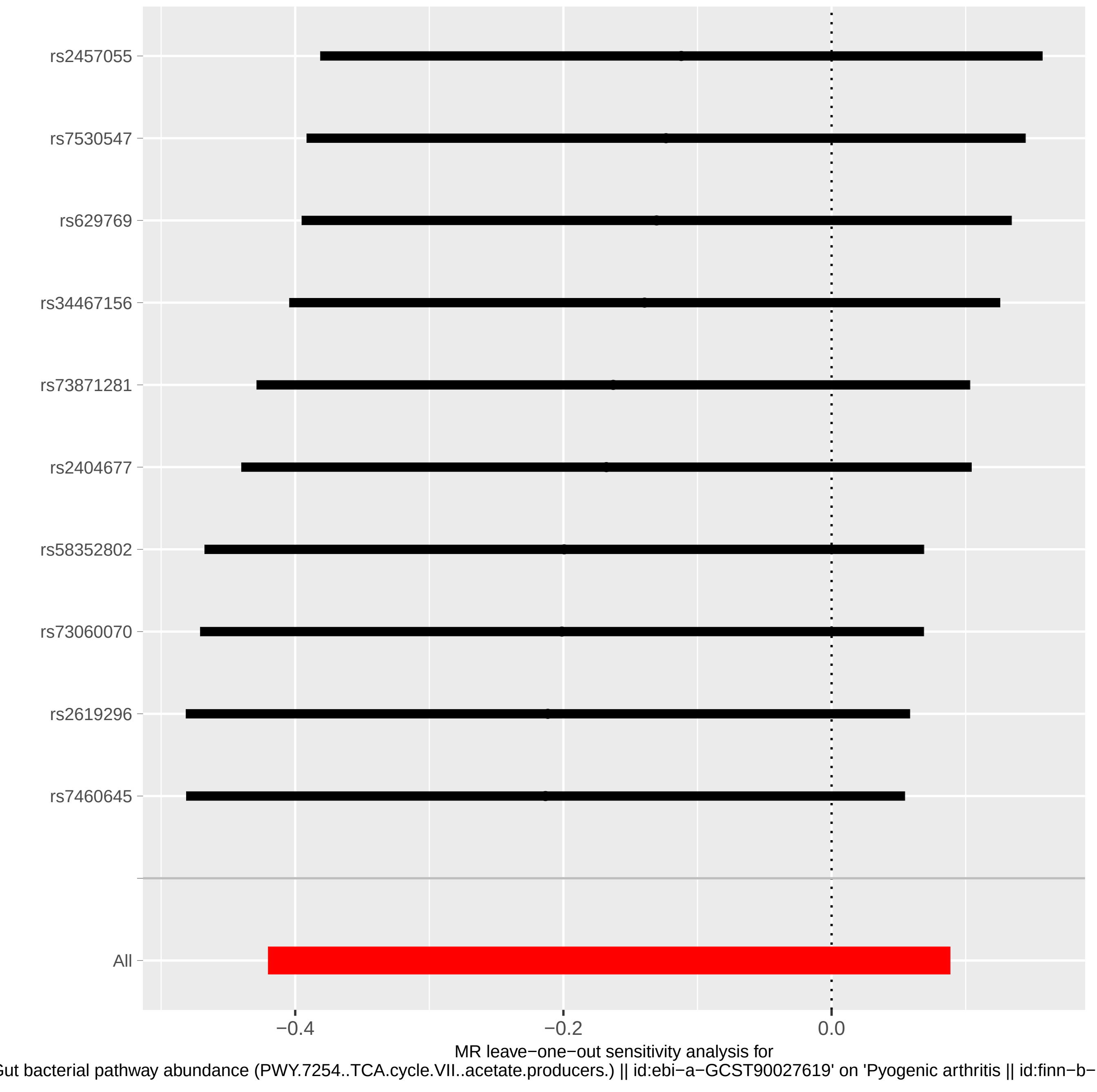
rs2457055
rs7530547
rs629769
rs34467156
rs73871281
rs2404677
rs58352802
rs73060070
rs2619296
rs7460645
All
−0.4
−0.2
0.0
MR leave−one−out sensitivity analysis for
Gut bacterial pathway abundance (PWY.7254..TCA.cycle.VII..acetate.producers.) || id:ebi−a−GCST90027619' on 'Pyogenic arthritis || id:finn−b−
